# A Cardiogenic Shock due to an Acute MI with LCA Arising from the Right Coronary Sinus Successfully Treated with PCI

**DOI:** 10.1155/2019/2018268

**Published:** 2019-07-04

**Authors:** Rayyan Jadeed, Klaus Pethig, Dirk Böcker

**Affiliations:** Department of Cardiology, St. Marien Hospital, Hamm, Germany

## Abstract

Coronary artery anomalies (CAAs) are a diverse group of congenital anomalies with an incidence ranging from 0.17% in autopsy cases to 1.2% in patients undergoing coronary angiography. The left coronary artery (LCA) originating from the right coronary sinus is a very rare CAA with a frequency of 0.03%. We present a very rare case of a cardiogenic shock as a consequence of an acute anterolateral myocardial infarction by a totally occlusive lesion in the long left main stem with a complete LCA arising from the right coronary sinus in an 85-year-old female. This lesion was successfully treated with 2 drug-eluting stents. This is perhaps the first published case about cardiogenic shock due to an acute myocardial infarction associated with this type of coronary anomalies, and it presents a special challenge in the catheter laboratory.

## 1. Introduction

Coronary artery anomalies (CAAs) are a diverse group of congenital anomalies with an incidence ranging from 0.17% in autopsy cases to 1.2% in patients undergoing coronary angiography [1, 2]. They are so interesting, as they form the second most frequent cause of sudden death in young athletes [3, 4]. It is reported that sudden death due to coronary arteries arising from the opposite sinus (ACAOS) is more frequent in young athletes <35 years than in older patients. It has been suggested that sudden death is less common in the latter group because of the stiffening of the aortic wall [5, 6].

We present a very rare case of a cardiogenic shock resulting from an acute myocardial infarction (MI) with a complete LCA arising from the right coronary sinus. With a frequency of 0.02%-0.05% on angiographic studies, this anomaly is one of the most uncommon coronary anomalies [2, 7].

## 2. Case Report

**Figure 1:**
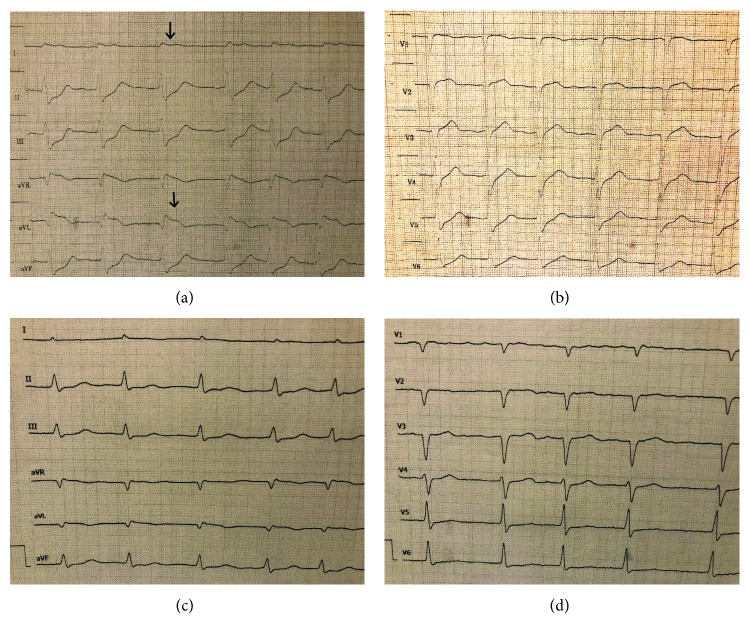
ECG findings: (a and b) ST elevations in leads I and aVL on admission. (c and d) After the procedure without elevations or significant depressions.

An 85-year-old female presented to the emergency department with a three-hour history of a typical anginal chest discomfort associated with shortness of breath, diaphoresis, nausea, and vomiting. The patient denied a history of coronary heart disease and was well anticoagulated for an atrial fibrillation. The patient was afebrile and her heart rate was 123 beats per minute with a blood pressure of 65/40 mmHg. Her oxygen saturation was 92% on 4 l/min via nasal cannula. On physical exam, she was in respiratory distress with a respiratory rate of 30 per minute and had no peripheral edema. On auscultation of the chest, she had diffuse bilateral crackles. The cardiac examination revealed an irregular rhythm without murmurs. Electrocardiogram (ECG) on presentation demonstrated ST elevations in leads I and aVL and ST depressions in leads II, III, aVF, V5, and V6 (Figures 1(a) and 1(b)).

Blood gas analysis (BGA) revealed increased lactate of 4.2 mmol/l.

**Figure 2:**
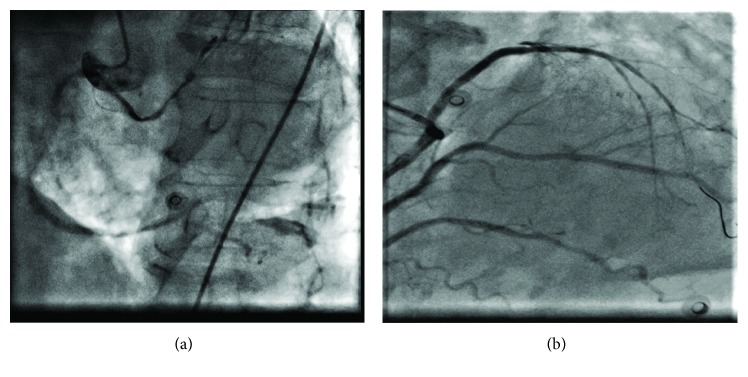
Angiographic findings: (a) RAO 40° view illustrates the left coronary artery with a 100% occlusive lesion in the distal LMCA and proximal LAD. (b) Cranial 30° view shows culprit vessel postintervention.

She underwent an emergency coronary angiography. The left main coronary artery (LMCA) could not be engaged with conventional diagnostic catheters for which an aortic root angiography using a pigtail catheter was performed and raised suspicion that the left coronary artery (LCA) was originating from the right coronary sinus. The injection of contrast in the right coronary sinus demonstrated an anomalous LCA separately arising from the right coronary sinus. The culprit lesion was a 100% occlusion in the distal LMCA and proximal left ascending artery (LAD) with grade 0 TIMI flow (Figure 2(a)). For the intervention, a 6F Amplatz right guide catheter (AR-1) was chosen, which engaged the LCA-ostium and provided an acceptable backup. Percutaneous intervention was performed with two drug-eluting stents achieving grade 3 TIMI postintervention without residual stenosis or complications (Figure 2(b)). The left coronary circumflex (LCX) was hypoplastic; therefore, it was ignored in the intervention strategy. The right coronary artery (RCA) was dominant and was divided at the crux of the heart into two large branches and continued posterolaterally as a large posterior lateral branch.

Postintervention ECG showed no significant ST elevations or ST depressions (Figures 1(c) and 1(d)).

During the procedure, a vasopressor was infused in small doses and multiple doses of intravenous diuretics were given.

After that, the patient was transferred to an intensive care unit; she did not require invasive ventilation. In two days, she was weaned off catecholamines and stepped down to the ward in good clinical status.

## 3. Discussion

The CAAs are classified into anomalies (1) of origin, (2) of courses, and (3) of termination.

The LCA originating from the right coronary sinus is a very unusual CAA.

It is difficult to engage the ostium in this anomaly, which makes an intervention in emergency settings particularly complex. The Amplatz right guide catheter seems to be suitable for this type of coronary anomalies.

To our knowledge, this is the first report of this anomaly with a cardiogenic shock related to a 100% occlusive lesion in the anomalous LCA successfully treated with percutaneous intervention (PCI). One previous case from Korea has described the same anomaly with an acute coronary syndrome by an up-to-90% occlusive lesion in the proximal LAD [8]. Shah et al. reported a similar case which was successfully treated with percutaneous intervention [9].

The clinical implications of CAAs vary depending on the type of anomalous artery and range, from ischemia presenting at an early stage of life to an incidental finding on angiography [10].

Coronary arteries arising from the opposite sinus (ACAOS), like the anomaly in our case, are the second leading cause of sudden cardiac death (SCD) in young athletes in the United States [11].

Although their incidence is low, CAAs should always be taken into consideration by clinicians due to their potentially fatal consequences.

Cardiologists in the catheter laboratory should have the required experience to intervene on such anomalous coronary arteries, especially in the situation of cardiogenic shock.
